# Dietary inflammatory index and objective disease activity in IBD: no association found

**DOI:** 10.1038/s41430-026-01713-6

**Published:** 2026-03-23

**Authors:** Rúbia Moresi Vianna de Oliveira, Ana Carolina Junqueira Vasques, Stefhani Andrioli Romero, Nitin Shivappa, Michael D. Wirth, James R. Hébert, Glaucia Fernanda Soares Ruppert Reis, Cristiane Kibune Nagasako

**Affiliations:** 1 Campinas State University, School of Medical Sciences, São Paulo, SP Brazil; 2 Campinas State University, School of Applied Sciences, São Paulo, SP Brazil; 3 Cancer Prevention and Control Program, University of South Carolina, Columbia, SC USA; 4 College of Nursing and Department of Epidemiology and Biostatistics, Arnold School of Public Health, Columbia, SC USA

**Keywords:** Inflammatory bowel disease, Health care

## Abstract

**Background:**

Inflammatory Bowel Disease (IBD) involves genetic and environmental factors, but the relationship between disease activity, adiposity, and diet remains unclear.

**Objective:**

To investigate the association between endoscopic/radiological activity of IBD, body adiposity, and the Dietary Inflammatory Index with or without adjustment for energy density (E-DII or DII).

**Method:**

An observational, cross-sectional study was carried out. Endoscopic activity was defined by an endoscopic Mayo score >2, Crohn’s Disease Endoscopic Index of Severity (CDEIS) > 5, and/or the presence of a deep ulcer in any intestinal segment. Body adiposity was estimated using the body mass index, waist circumference, and waist-hip ratio (WHR). The DII and E-DII scores were calculated from a validated quantitative food frequency questionnaire. According to the DII and E-DII, the patients were divided into three groups: the first with the least pro-inflammatory diet and the third with a predominantly pro-inflammatory diet.

**Results:**

Of the 62 patients, 58.1% (*n* = 36) were in remission (RD) and 41.9% (*n* = 26) had active disease (AD). The proportion of patients with overweight/obesity was 69.4% (*n* = 25) in the RD group and 50.0% (*n* = 13) in the AD group. Patients in remission exhibited significantly higher WHR (*p* < 0.05) and a greater frequency of central obesity (*p* < 0.01). A predominantly pro-inflammatory diet was common across both groups; 58.3% (*n* = 21) of RD patients and 50.0% (*n* = 13) of AD patients were in the highest DII tertile. Similar results were found for the E-DII.

**Conclusions:**

Among patients with IBD, pro-inflammatory dietary patterns and excess adiposity are highly prevalent. Despite greater central adiposity in patients in remission, no significant associations were found between DII or EDII scores and endoscopic and radiological markers of disease activity.

## Introduction

Inflammatory bowel disease (IBD), which includes ulcerative colitis (UC) and Crohn’s disease (CD), is characterized by chronic, immune-mediated gut inflammation [1]. This condition results from a complex interaction between genetic susceptibility and environmental factors that influence the immune response [2–5].

Gastrointestinal disorders have stimulated new research into the relationship between dietary patterns and IBD [6, 7]. Several studies support a connection between a Western diet and adiposity, which may favor the production of inflammatory cytokines and alter the gut microbiota [8–13]. Studies show that high body adiposity in patients with IBD could impair drug treatment and induce inflammation. However, data regarding the role of overweight and obesity in IBD remains discordant. While historically IBD was considered a malnourishing condition, recent data show a rising prevalence of obesity in this population, mirroring trends in the general population [14–16]. Some studies suggest that excess adiposity may lead to a more severe disease course and impair response to biologic therapies [17, 18]. Conversely, other studies have failed to find a significant association or have even described a potential protective effect in specific subgroups, a phenomenon known as the ‘obesity paradox’ in IBD [19].

More recent evidence indicates that diet also plays a key role in regulating chronic inflammation through various mechanisms [20, 21]. Diet quality indices are increasingly used in epidemiological studies to represent dietary patterns. These standards can overcome potential confusion related to specific nutrients or foods, as the wide variety of foods in a diet results in a significant diversity of nutrients that can interact synergistically or antagonistically [22].

In this context, the Dietary Inflammatory Index (DII) was developed by Shivappa et al. [21] to characterize the inflammatory potential of an individual’s diet. It has been widely used to investigate the relationship between diet and non-communicable diseases, including obesity, cancer, depression, and cardiometabolic, respiratory, and musculoskeletal disorders [23]. Although an environmental factor linked to IBD is the Western diet, characterized by high fat and sugar content, as well as decreased consumption of fruits and vegetables [24], the relationship between the inflammatory activity of IBD and dietary patterns remains unclear. To date, few studies have assessed the correlation between endoscopic and radiological activity in IBD and body composition [25–28]. Therefore, this study aimed to investigate the association between endoscopic and radiological activity of IBD and DII, as well as body adiposity.

## Materials and methods

### Study design

This observational cross-sectional study included patients with IBD (including both CD and UC) from the outpatient gastroenterology clinic of the tertiary hospital in Brazil, from March 2021 to March 2022.

**Fig. 1:**
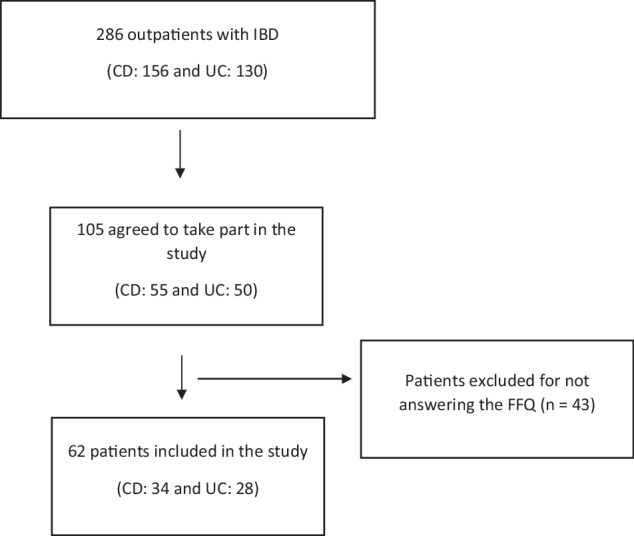
Patient inclusion flowchart. CD Crohn’s disease, UC ulcerative colitis, FFQ food frequency questionnaire.

This study included adult patients aged 18 years or older with a confirmed diagnosis of CD or UC, as determined by clinical, endoscopic, imaging, and/or histopathological criteria. Patients with no confirmed diagnosis, pregnant women, patients undergoing nutrition therapy, and individuals with neurological diseases that affect cognition and the ability to answer questions were excluded. The patient inclusion flowchart is described in Fig. 1.

### Ethical aspects

Written informed consent was obtained from each subject. This study protocol was approved by the Ethics and Research Committee of our university (CAAE no. 39030520.8.0000.5404).

### Clinical assessment

We reviewed medical records and collected sociodemographic information (age and gender); lifestyle habits (current or previous smoking and alcohol consumption); other comorbidities (arterial hypertension [SAH], DM, dyslipidemia); clinical data related to the disease, including total illness duration, extra-intestinal manifestations, current treatment (corticosteroids, salicylic derivatives, immunosuppressant and/or immunobiological drugs), and previous surgeries and hospitalizations due to IBD.

### Assessment of endoscopic and radiological activity in IBD

Endoscopic activity in CD was assessed by colonoscopy, nuclear magnetic resonance scan, or enterography performed in the last 6 months [29]. Disease activity in CD was defined by colonoscopy with a Crohn’s Disease Endoscopic Index of Severity (CDEIS) score of 5 or higher, or radiological strata indicating the presence of a deep ulcer in at least one intestinal segment. Patients who did not meet these criteria were considered in endoscopic remission.

Patients with UC were classified according to the Mayo endoscopic score, distinguishing between active disease (Mayo score 2 or 3) and remission (Mayo score 0 or 1) [30].

### Assessment of anthropometric parameters

Body mass index (BMI) was calculated as weight in kilograms divided by height in meters squared. Weight adjustments were made for edema, considering the dry weight without edema; when peripheral edema was present, BMI and weight variation calculations were performed using the patient’s dry weight, defined as the euvolemic weight determined by clinical examination during the consultation [3]. BMI was assessed by using WHO criteria: <18.5 kg/m² - malnutrition; ≥18.5–24.9 kg/m² - eutrophic; ≥25–29.9 kg/m² - overweight; and ≥30 kg/m² - obesity [31, 32]. For analysis, patients were divided into two groups: malnutrition/eutrophic and overweight/obesity.

The waist-to-hip ratio (WHR) was used to estimate the deposition of central adiposity. Waist circumference was measured using a non-extensible measuring tape at the midpoint between the lowest rib and the iliac crest. The presence of central obesity was based on waist circumference (>88 cm in women and >102 cm in men) [33]. For hip circumference, the measurement was taken at the level of the greater trochanter of the femur. Measurements were taken three times, and mean values were used. The ratio was established by dividing waist circumference by hip circumference and was considered high when WHR > 0.90 in men and > 0.80 in women [34, 35].

### Assessment of dietary intake

A single registered dietitian conducted individual interviews with all participants. Dietary intake was assessed using a validated, quantitative food frequency questionnaire (FFQ) with 60 items, where consumption frequencies ranged from 0 to 10 times per day, week, month, or year. Portion sizes were categorized as small, medium, large, and extra-large, and participants were guided using a photographic album depicting household measures to accurately estimate portion sizes [36, 37]. Reported frequencies and portion sizes were converted into grams. Daily intakes of energy, nutrients, and bioactive compounds were calculated using data primarily from the Brazilian Food Composition Tables [38]. When data were unavailable, the U.S. Department of Agriculture database was consulted [39]. The Brazilian Carotenoid Composition Table in Foods [40] and the Phenol-Explorer database [41] were used to estimate β-carotene and flavonoids, respectively.

### Dietary inflammatory index

The inflammatory profile of the diet was evaluated using the DII calculation developed by Shivappa et al. [21]. To calculate the DII and energy-adjusted DII (E-DII) score, dietary information for 39 food components (energy, protein, carbohydrates, total fiber, fat, linoleic and linolenic fatty acids, saturated fatty acids, cholesterol, trans fatty acids), mono and polyunsaturated fatty acids, iron, selenium, magnesium, zinc, vitamins A, C, D, E, B12, B1, B2, B3, B6 and B9, alcohol, caffeine, beta-carotene, eugenol, flavan-3-ol, flavanols, flavones, flavanones, anthocyanidins, isoflavones, garlic, onion and tea) from each participant were registered.

The inflammatory potential of each dietary parameter was evaluated separately, and the value obtained was converted into a percentile score. A scoring algorithm sums all inflammatory indices for each dietary parameter, based on individual intake values, and calculates each individual’s DII. In this study, DII was calculated with and without adjustment for dietary energy density (E-DII). The DII, therefore, varies for each individual; the higher the value obtained when calculating the DII or E-DII, the more pro-inflammatory the diet presented by the patient.

For comparison between endoscopic and radiological activity and remission groups, patients were divided according to DII and E-DII into three groups, based on the DII or E-DII value, where the first (DII: −3.50–0.04/E-DII: −1.37–−0.26) is characterized by a more anti-inflammatory diet and the third (DII: 1.12–3.49/E-DII: 1.08–4.55) by a predominantly pro-inflammatory diet.

### Statistical analysis

Categorical variables were represented in frequency tables and percentages (%), and quantitative variables were presented as mean ± standard deviation, along with median values (including minimum and maximum values). Groups were compared using the Chi-square test or Fisher’s exact test for categorical variables and the Mann–Whitney test for numeric variables. The significance level adopted for the study was 5%. Statistical analyses were performed using the SAS (Statistical Analysis System) System for Windows, version 9.4.

## Results

### Overall characteristics of participants

A total of 62 patients were included in this study. The average age was 47.8 ± 15.2 years, and 64.5% were female. A small portion had active smoking (6.5%) and alcohol consumption (3.2%). Assessing other comorbidities, 32.3% had SAH, 17.7% had DM, and 46.8% had dyslipidemia.

Of the total sample, 54.8% had CD and 45.2% had UC, with an average disease duration of 13 years. Indeed, 27.4% of patients had some extraintestinal manifestation related to IBD. Current treatment, need for hospitalization, and surgery in the last 6 months due to IBD were also evaluated (Table 1).

**Table 1:** Clinical and demographic characteristics of patients with IBD in endoscopic and radiological activity and remission.

Characteristic	AD (*n* = 26)	RD (*n* = 36)	Total (*n* = 62)	*P*-value
Age (years)	43.3 ± 14.2	51.0 ± 15.2	47.8 ± 15.2	**0.0413**
Female gender, *n* (%)	14 (53.8)	26 (72.2)	40 (64.5)	0.1356
Smoking, *n* (%)	0 (0)	4 (11.1)	4 (6.5)	0.1324
Alcohol consumption, *n* (%)	1 (3.8)	1 (2.8)	2 (3.2)	0.6295
SAH, *n* (%)	5 (19.2)	15 (41.7)	20 (32.3)	0.0622
DM, *n* (%)	4 (15.4)	7 (19.4)	11 (17.7)	0.7479
Dyslipidemia, *n* (%)	11 (42.3)	18 (50)	29 (46.8)	0.5492
IBD (CD/UC), *n* (%)	13 (50)/13 (50)	21 (58.3)/15 (41.7)	34 (54.8)/28 (45.2)	0.5153
Illness duration (years)	9.9 ± 8.7	15.3 ± 10.3	13.0 ± 10.0	**0.0184**
Extra-intestinal disease, *n* (%)	5 (19.2)	12 (33.3)	17 (27.4)	0.2193
Current treatment:				
- corticosteroid, *n* (%)	2 (7.7)	1 (2.8)	3 (4.8)	0.5669
- salicylic derivative, *n* (%)	5 (19.2)	7 (19.4)	12 (19.4)	0.9832
- immunosuppressant, *n* (%)	16 (61.5)	18 (50.0)	34 (54.8)	0.3677
- immunobiological, *n* (%)	15 (57.7)	16 (44.4)	31 (50.0)	0.3033
Hospitalization due to IBD in the last 6 months, *n* (%)	6 (23.1)	7 (19.4)	13 (21.0)	0.7288
Surgery due to IBD in the last 6 months, *n* (%)	6 (23.1)	9 (25.0)	15 (24.2)	0.8615
Overweight/obesity, *n* (%)	13 (50)	25 (69.4)	38 (61.3)	0.1209
High waist-to-hip ratio, *n* (%)	18 (69.2)	35 (97.2)	53 (85.5)	**0.0029**
Central obesity, *n* (%)	8 (30.8)	23 (63.9)	31 (50)	**0.0101**

*AD* active disease, *RD* remission disease, *IBD* inflammatory bowel disease, *CD* Crohn’s disease, *UC* ulcerative colitis, *SAH* systemic arterial hypertension, *DM* diabetes mellitus.

Those p-values that were statistically significant were highlighted in bold.

In the anthropometric assessment, most patients (61.3%) were classified as having overweight or obesity. Additionally, 85.5% exhibited a high waist-to-hip ratio (WHR), and 50% showed central obesity as indicated by an increased waist circumference.

### Endoscopic and radiological activity in IBD

For comparison purposes, the groups were divided based on endoscopic and/or radiological activity (*n* = 26, 41.93%) and remission (*n* = 36, 58.07%) criteria. The activity group was younger (43.1 ± 14.2 *versus* 51 ± 15.2 years, *p* = 0.04) and had a shorter illness duration (9.9 ± 8.7 *versus* 15.3 ± 10.3 years, *p* = 0.01) compared to the remission group. There were no differences between the groups about gender, smoking, alcohol consumption, other comorbidities (SAH, DM, and dyslipidemia), IBD distribution, whether CD or UC, presence of extra-intestinal manifestations, current treatment, and prior hospitalization or surgery due to IBD (Table 1).

In the anthropometric assessment, overweight or obesity was more frequent in the remission group: 25 (69.4%) *versus* 13 (50%); as was a high waist-to-hip ratio: 97.2% *versus* 69.2% (*p* = 0.0029) and central obesity: 63.9% *versus* 30.8% (*p* = 0.01).

### Dietary intake and inflammatory potential of the diet

**Fig. 2:**
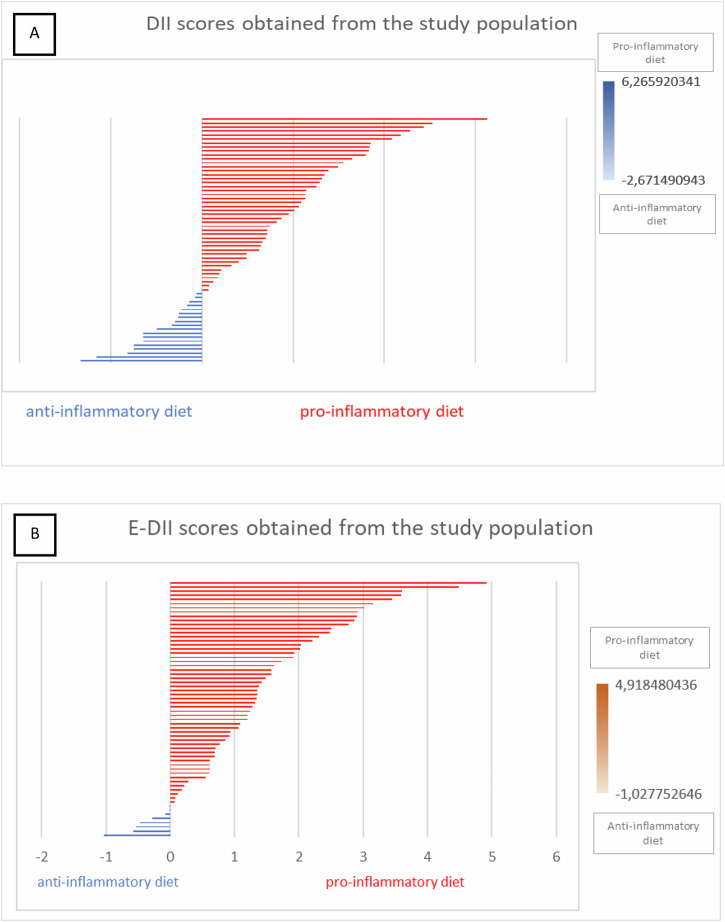
Distribution of dietary inflammatory index scores in patients with inflammatory bowel disease. **A** Distribution of crude dietary inflammatory index (DII) scores in the study population. **B** Distribution of energy-adjusted dietary inflammatory index (E-DII) scores in the study population. DII dietary inflammatory index, E-DII energy-adjusted dietary inflammatory index.

In the total study population, a predominance of a pro-inflammatory diet was observed, as the average DII score was positive [1.31 ± 1.97 (−2.32 – When stratifying patients into tertiles based on IBD and EDII scores, we found that most individuals in this cohort clustered in the highest tertile for both IBD (54.8%) and E-IBD (56.5%), supporting a more pro-inflammatory dietary profile. 5.06)]. The same was observed for E-DII, with an average value of +1.36 in the population (Fig. 2).

Both the AD (50%) and RD (58.3%) groups were predominantly classified in the third tertile, indicating a more pro-inflammatory dietary pattern. Similarly, for E-IBD, 57.7% of participants in the AD group and 55.6% in the RD group were also in the highest inflammatory tertile (Table 2).

**Table 2:** Evaluation in tertiles of DII and E-DII in patients with IBD in endoscopic and radiological activity and remission.

Parameter	AD (*n* = 26)	RD (*n* = 36)	Total (*n* = 62)	*P*-value
DII (%)				0.77
−3.50–0.04	8 (30.8)	10 (27.8)	18 (29.0)	
0.05–1.11	5 (19.2)	5 (13.9)	10 (16.1)	
1.12–3.49	13 (50)	21 (58.3)	34 (54.8)	
E-DII (%)				0.18
−1.37–−0.26	4 (15.4)	1 (2.8)	5 (8.1)	
−0.27–1.08	7 (26.9)	15 (41.7)	22 (35.5)	
1.09–4.55	15 (57.7)	20 (55.6)	35 (56.5)	

*DII* dietary inflammatory index, *E-DII* energy-adjusted dietary inflammatory index, *AD* active disease, *RD* remission disease, *IBD* inflammatory bowel disease.

Assessment of IID and Energy, as well as micro- and macronutrient intakes, did not differ significantly according to disease activity (Supplementary material: Supplementary Table S1).

## Discussion

**Fig. 3:**
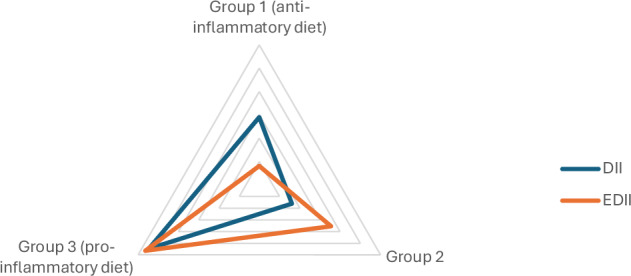
Evaluation in group of DII and E-DII in all IBD patients. DII dietary inflammatory index, E-DII energy-adjusted dietary inflammatory index, Group 1 more anti-inflammatory diet, Group 2 middle group, Group 3 more pro-inflammatory diet.

In this cross-sectional study, we investigated the relationship between dietary inflammatory potential, body adiposity, and objective markers of disease activity in a cohort of Brazilian IBD patients (Fig. 3). Our main findings were a high prevalence of both overweight/obesity and pro-inflammatory dietary patterns, yet no significant association was found between the DII/E-DII and endoscopic or radiological disease activity.

A significant observation was the high prevalence of overweight/obesity (61.3%), especially among individuals in the remission group. This rate exceeds the 50.3% reported in a large Brazilian multicenter cohort of 1703 individuals with IBD patients (33.1% overweight; 17.2% obesity), which also found abdominal obesity in 22.6% of patients, particularly among women, as determined by waist circumference [42]. In our cohort, central obesity was present in 50% and an elevated WHR in 85.5%, indicating an even greater burden of visceral adiposity. These discrepancies may be attributed to differences in patient age distribution, disease duration, regional dietary habits, or the use of corticosteroids, which are known to promote central fat accumulation [43]. Additional studies suggest that patients with IBD are increasingly reflecting the weight gain patterns seen in the general population worldwide. For example, a U.S. tertiary-center study found that 46.7% of patients with Crohn’s disease were overweight or obese at the time of diagnosis [44]. Similarly, a nationwide Korean analysis conducted from 2010 to 2019 reported a continuous increase in BMI and obesity prevalence among patients with both Crohn’s disease and ulcerative colitis [45]. This finding is consistent with emerging evidence that challenges the traditional perception of IBD as a predominantly wasting disease and underscores the growing recognition of obesity as a relevant comorbidity in this population [46].

In our study, overweight/obesity, elevated WHR, and central obesity were more prevalent in the remission group. Additionally, the remission group is characterized by older age and a higher prevalence of metabolic comorbidities, as well as lower levels of physical activity.

While visceral fat is metabolically active and a source of pro-inflammatory cytokines, its clinical impact on IBD activity remains controversial [47]. Some studies associate obesity with more severe disease and reduced biological persistence, particularly for fixed-dose anti-TNF agents [48, 49].

In contrast, other studies have reported neutral effects or even protective associations in certain patient subgroups [50–56]. These heterogeneities may partly explain why, in our sample, greater adiposity in the remission group did not translate into higher objective inflammatory activity. The higher rates of central adiposity in the remission group are intriguing and warrant further investigation, as visceral fat is a known source of pro-inflammatory cytokines [57, 58].

We observed positive mean DII and E-DII scores, with a predominance of patients in the most pro-inflammatory tertile across the cohort, and no differences between patients with active disease and those in remission. These findings are consistent with previous studies, which have reported that patients with IBD tend to consume diets high in ultra-processed foods, saturated fats, and added sugars, while having low intakes of fruits and vegetables. Such a dietary pattern, characteristic of a Westernized diet, is linked to gut dysbiosis and increased intestinal permeability. A 2025 systematic review and meta-analysis further concluded that pro-inflammatory diets increase the risk of ulcerative colitis [59]. Additionally, a narrative review highlighted plausible mechanistic overlaps between obesity-promoting diets and the pathogenesis of IBD, especially Crohn’s disease [60]. Our results therefore reinforce the growing body of evidence connecting poor overall diet quality with IBD.

However, the literature on associations between the DII and IBD activity remains heterogeneous. Some cross-sectional studies, such as those by Lamers et al. [61], have found positive correlations between higher DII scores and active Crohn’s disease, but not ulcerative colitis. In contrast, Mirmiran et al. [55] reported no association between DII and clinical activity, while Bakhtiari et al. [62] observed higher DII scores in patients with active ulcerative colitis compared to those in remission. In addition, recent multicenter cohorts have linked pro-inflammatory diets to elevated fecal calprotectin and greater risk of clinical relapse, potentially mediated by alterations in gut microbiota—such as increased abundance of Veillonella spp. [63]

Recent reviews [64, 65] highlight that while anti-inflammatory dietary patterns (e.g., Mediterranean-style diets) appear to lower disease risk and possibly attenuate inflammation, the relationship with objective activity measures such as endoscopic or radiologic scores is less consistent.

The lack of a link between DII/E-DII and disease activity in our study should be viewed cautiously. This cross-sectional design limits the ability to establish causality, as dietary effects on inflammation typically develop over time and may not be apparent at a single point. The small sample size (*n* = 62) may have also reduced the power to detect modest associations. Additionally, the predominance of pro-inflammatory dietary patterns in both groups likely reduced variability, making it more difficult to identify significant differences. Finally, using endoscopic and radiological data from the past six months may not accurately represent inflammatory activity at the time of dietary assessment, potentially leading to misclassification of disease activity and weakening the observed associations. On the other hand, this study has several strengths, such as the use of objective endoscopic and radiological criteria to assess disease activity, which are more reliable than symptom-based clinical scores. Additionally, employing a validated Food Frequency Questionnaire (FFQ) tailored to Brazilian dietary habits, along with data collection by a single, trained registered dietitian, enhances the internal validity of our dietary assessment.

This is the first Brazilian study to investigate the relationship between DII/E-DII and objective endoscopic/radiologic activity in IBD, categorized according to CDEIS/Mayo scores and standardized radiologic criteria. Despite the lack of association, the high prevalence of central adiposity and the widespread pro-inflammatory dietary profile underscore the urgent need for structured dietary counseling and weight management in IBD care. Dietary interventions promoting anti-inflammatory patterns, such as the Mediterranean diet or IBD-AID, may be particularly beneficial not only for gut inflammation but also for reducing the burden of cardiometabolic comorbidities and potentially improving therapeutic persistence [60].

Future studies should adopt prospective designs with repeated nutritional assessments, include objective and centralized activity scoring, and integrate advanced body composition analysis alongside biomarker profiling. Incorporating metabolomic signatures of dietary intake and a detailed evaluation of ultra-processed food consumption could help elucidate mechanistic pathways linking diet, adiposity, and mucosal inflammation. Pragmatic nutritional intervention trials, testing anti-inflammatory dietary patterns as adjuvant therapy, are warranted to determine causal effects and clinical applicability.

## Conclusion

In conclusion, our study did not identify a significant association between the inflammatory potential of the diet and objective markers of disease activity in this cohort of patients with IBD. Future research should prioritize larger, prospective longitudinal studies to elucidate better the long-term effects of dietary patterns on disease trajectory and to investigate further the interactions between diet, the gut microbiome, and host genetics.

### Reporting summary

Further information on research design is available in the Nature Research Reporting Summary linked to this article.

## Supplementary information

Reporting Summary

Table S1

## Data Availability

The data supporting the findings of this study are available upon request from the corresponding author, RMVO. The data is not publicly available because it contains information that could compromise the privacy of research participants.
