# Supplementary material for: Dietary inflammatory index and objective disease activity in IBD: no association found
Source: Eur J Clin Nutr. 2026 Mar 23;80(5):491–7. doi: 10.1038/s41430-026-01713-6 (PMC13186691; doi:10.1038/s41430-026-01713-6)
Supplement: Supplementary file 2 — Table S1 [file 41430_2026_1713_MOESM2_ESM.pdf]

Table S1: Clinical and demographic characteristics of patients with IBD in endoscopic and radiological activity and remission.

**Characteristic**	**AD (n = 26)**	**RD (n= 36)**	**Total (n = 62)**	**P-value**
Age (years)	43.3 ± 14.2	51.0 ± 15.2	47.8 ± 15.2	**0.0413**
Female gender, n (%)	14 (53.8)	26 (72.2)	40 (64.5)	0.1356
Smoking, n (%)	0 (0)	4 (11.1)	4 (6.5)	0.1324
Alcohol consumption, n (%)	1 (3.8)	1 (2.8)	2 (3.2)	0.6295
SAH, n (%)	5 (19.2)	15 (41.7)	20 (32.3)	0.0622
DM, n (%)	4 (15.4)	7 (19.4)	11 (17.7)	0.7479
Dyslipidemia, n (%)	11 (42.3)	18 (50)	29 (46.8)	0.5492
IBD (CD/UC), n (%)	13 (50) / 13 (50)	21 (58.3) / 15 (41.7)	34 (54.8) / 28 (45.2)	0.5153
Illness duration (years)	9.9 ± 8.7	15.3 ± 10.3	13.0 ± 10.0	**0.0184**
Extra-intestinal disease, n (%)	5 (19.2)	12 (33.3)	17 (27.4)	0.2193
Current treatment:<br>- corticosteroid, n (%)	2 (7.7)	1 (2.8)	3 (4.8)	0.5669
- salicylic derivative, n (%)	5 (19.2)	7 (19.4)	12 (19.4)	0.9832
- immunosuppressant, n (%)	16 (61.5)	18 (50.0)	34 (54.8)	0.3677
- immunobiological, n (%)	15 (57.7)	16 (44.4)	31 (50.0)	0.3033
Hospitalization due to IBD in the last 6 months, n (%)	6 (23.1)	7 (19.4)	13 (21.0)	0.7288
Surgery due to IBD in the last 6 months, n (%)	6 (23.1)	9 (25.0)	15 (24.2)	0.8615
Overweight / obesity, n (%)	13 (50)	25 (69.4)	38 (61.3)	0.1209
High waist-to-hip ratio, n (%)	18 (69.2)	35 (97.2)	53 (85.5)	**0.0029**
Central obesity, n (%)	8 (30.8)	23 (63.9)	31 (50)	**0.0101**

AD: active disease; RD: remission disease: IBD: inflammatory bowel disease; CD: Crohn’s disease; UC: ulcerative colitis; SAH: systemic arterial hypertension; DM: diabetes mellitus
